# Rate and associated factors of refusal to perform immunochemical Faecal Occult Blood Test (iFOBT) among semi-urban communities

**DOI:** 10.1371/journal.pone.0258129

**Published:** 2021-10-07

**Authors:** Sharifah Saffinas Syed Soffian, Nazarudin Safian, Azmawati Mohammed Nawi, Shahrul Bariyah Ahmad, Huan-Keat Chan, Muhammad Radzi Abu Hassan

**Affiliations:** 1 Department of Community Health, Faculty of Medicine, Universiti Kebangsaan Malaysia, Kuala Lumpur, Malaysia; 2 Public Health Division, Kedah State Health Department, Kedah, Malaysia; 3 Clinical Research Center, Sultanah Bahiyah Hospital, Kedah, Malaysia; Unaizah College of Pharmacy, Qassim University, SAUDI ARABIA

## Abstract

The uptake of the immunochemical faecal occult blood test (iFOBT) in many countries with an opportunistic colorectal cancer (CRC) screening programme remains suboptimal. This study aimed to determine the rate, associated factors and reasons of refusal to perform the iFOBT test offered under an opportunistic CRC screening programme in semi-urban communities. This cross-sectional study was conducted among the average-risk individuals living in semi-urban areas, who sought care from public primary care centres across Kedah state, Malaysia. The information regarding the sociodemographic and clinical characteristics of individuals who were offered the iFOBT between January and April 2019, along with their willingness to perform the test, was gathered. The factors associated with the refusal were further explored using the logistic regression analysis. The individuals offered the iFOBT (n = 920) were mainly female (52.4%) and had a mean age of 58.7±10.6 years. The refusal rate of the iFOBT was 32.2%. Patients who did not have hypertension (adjusted OR: 3.33; 95% CI: 2.44, 4.54), did not have CRC symptoms (adjusted OR: 3.15; 95% CI:1.26, 7.89), had the test offered by either medical assistants (adjusted OR: 2.44; 95% CI: 1.71, 3.49) or nurses (adjusted OR: 2.41; 95% CI 1.65, 3.51), did not have diabetes (adjusted OR: 1.99; 95% CI: 1.42, 2.77),and were not active smokers (adjusted OR: 1.74; 95% CI: 1.22, 2.47), were more likely to refuse the iFOBT. The common reasons of refusing the test included “feeling not ready for the test” (21.6%) and “feeling healthy” (14.9%). The iFOBT was refused by one-third of the average-risk individuals from semi-urban communities. The associated factors and reasons of refusal found in this study could guide policymakers in developing targeted interventions to boost the uptake of CRC screening in Malaysia.

## Introduction

More than 1.9 million colorectal cancer (CRC) cases were recorded in 2020 alone [1], accounting for 10% of the new cancer cases diagnosed in the same year. CRC has long been identified as one of the most common cancer types and a major leading cause of deaths [2]. Although an improvement in the overall 5-year survival rate of CRC patients from 48% to 71% was recorded between 1995 and 2014 [3], the geographical variations in the incidence and mortality of CRC, mainly due to the differences in the levels of economic development and culture, have been widely reported.

As reported in the GLOBOCAN report in 2018, CRC is the third most prevalent cancer after lung and breast cancer, of which the cancer trend varies depending on the socioeconomic level of the country [2]. It is also the second leading cause of death among all types of cancer in both men and women. While countries with a medium to high Human Development Index (HDI) showed a constant increase in CRC incidence throughout the last decade, countries with the highest HDI, such as the United States witnessed a reduction in the CRC incidence among individuals above 50 years of age, mainly due to the early detection of tumors [4]. Meanwhile, more than two-thirds of the CRC patients in countries with a medium to high HDI presented to health facilities for care only at advanced stages of the disease [4–6]. Generally, the public awareness of CRC and the need for early screening remains poor worldwide, very often resulting in delayed diagnosis of the disease [7–10].

The delayed presentation of CRC patients for medical care is critical, particularly in semi-urban communities, as suggested by their lower 5-year survival rate as compared with those living in urban areas [11,12]. As the treatment for advanced CRC is generally more costly, more budget has been allocated to the public health system to scale up the screening in average-risk individuals [12,13]. More so to include the expenditure made by those who have been seeking care from private settings in places where universal health coverage not applied. While the global population is shifting towards an ageing population, the CRC burden, along with the concomitant financial burden, is projected to constantly grow constantly.

However, it is also well known that the survival of CRC patients can be improved through early detection and timely treatment, primarily by removing premalignant adenomatous polyps and localized tumors. A study from Norway suggests that CRC screening can potentially reduce the mortality of patients by 7%, and timely treatment is likely to further prevent 12% of CRC-related deaths [14]. A structured, government-led CRC screening programme is also likely to lower the mortality rate by at least 30%, as demonstrated in Japan and China [15,16].

As a strategy to encourage the early detection of CRC, an opportunistic screening programme using the immunochemical fecal occult blood test (iFOBT) kit, a stool-based screening test kit, has long been implemented in many countries. The results of the iFOBT are often used to guide clinicians in evaluating the need for colonoscopy. The US Preventive Task Force recommends the iFOBT to be used for average-risk individuals, who are aged between 50 and 75 years and do not have a family history of CRC or other cancer types [17]. The test is normally offered to patients by clinicians, and occasionally by allied health professionals.

Despite the availability of alternatives, including blood- and urine-based biomarkers, the iFOBT remains a widely accepted and yet one of the most cost-effective screening tests [18–20]. However, its uptake has been found to be suboptimal over the years, particularly in the semi-urban communities [9,21,22]. This study was designed to determine the rate and associated factors of refusal to perform the iFOBT among the average-risk individuals from the semi-urban communities.

## Materials and methods

This study was conducted in Kedah, a state located in northern Malaysia with an urbanisation level below 70% [23]. In tandem with the spurt of urban population growth, most of the healthcare facilities, including the private medical health institutions, were more concentrated in the major cities. Generally, the semi-urban communities in Malaysia are highly dependent on public health clinics for health services. The public primary care centres are well distributed across the Kedah state, providing free-of-charge medical services to a population of 2.1 million. More than 80% of the residents in the state belongs to the bottom 40% (B40) income group [24], who earn a living from agricultural activities with a monthly income below MYR3000 [25]. While medical resources disproportionately benefit those living in urban areas, the semi-urban population are therefore often neglected.

**Fig 1:**
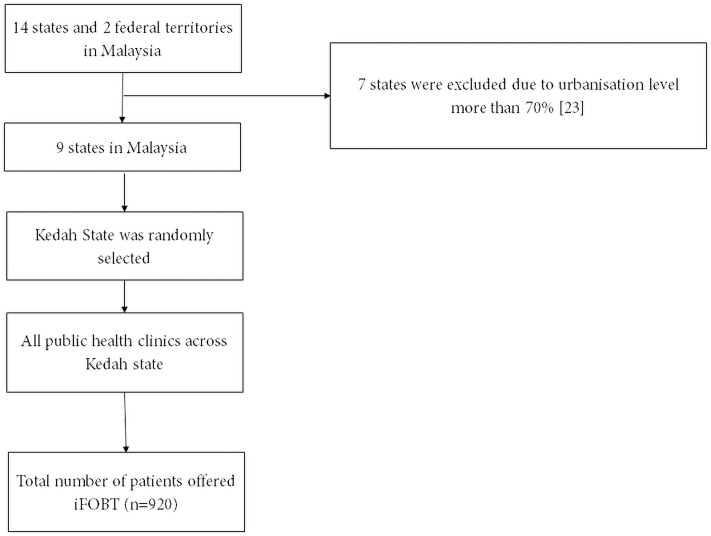
Study flow chart.

This cross-sectional study was conducted in the 51 public primary care centers within the state of Kedah which was randomly selected from 9 states that have urbanisation level below 70% [23] (Fig 1). The permission to perform the study was sought from the Kedah State Health Department, and the study proposal (NMRR-19-94-45685) was approved by the Medical Research and Ethics Committee, Ministry of Health Malaysia. Individuals aged 18 years and above, who sought care from any of the primary care centers and were offered the iFOBT between 1^st^ January 2019 and 30^th^ April 2019, were included in this study. The recruited sample was representative of the major population due to the high attendance to the public primary care centers, accounting to more than 150 million patients for diabetic alone as recorded in the National Diabetes Registry [26].

A questionnaire was developed to collect information on the socio-demographics of the respondents and explore the reasons for refusal. A content validation was performed by two public health experts. The information gathered from the recruited individuals included their socio-demographics (age, gender, ethnicity, education level and employment status), body mass index (BMI), smoking status, comorbidities (hypertension, diabetes mellitus, dyslipidemia), family history of cancer, experience with the symptoms of CRC (abdominal pain, altered bowel habit and bloody stool), and reasons for refusing the iFOBT if they did. The data collection was performed by healthcare providers stationed at the health clinics, either clinicians, medical assistants, or nurses, at the time when they offered the iFOBT. Patients who consented to perform the test were then instructed to bring home a container, which were to be returned to the clinics within three days following the stool collection for subsequent laboratory-based assessment. Verbal consent obtained from participants were documented in the patient medical records kept at the health clinics.

The data analysis was performed using the SPSS version 21.0 for Windows (IBM, New York). All categorical variables were summarised as frequencies and percentages, and the numerical variables as means and standard deviations (SDs). The factors associated with the refusal of the iFOBT test were also explored using the backward stepwise logistic regression analysis, with the results presented as odds ratios (ORs) and 95% confidence intervals (CIs). The final model was also tested for interaction and multicollinearity, and its fitness was confirmed by using the Hosmer–Lemeshow goodness-of-fit test.

## Results

Over the stipulated four-month period, a total of 920 individuals were offered the iFOBT test. They were mainly female (52.4%) and of Malay ethnicity (79.0%), with a mean age of 58.7 (SD = 10.6 years). More than 50% of them had at least a secondary education level (56.5%). However, approximately half of them were unemployed at the time when the iFOBT was offered. Slightly half of them were either overweight or obese, and nearly one-fifth of them were active smokers. Approximately one in every two, one in every three and one in every four of them were also, respectively, found to have hypertension, diabetes mellitus and dyslipidaemia. More than 90% of them reported neither a family nor a personal history of cancer. The symptoms of CRC were present in only 4.1% of them. The iFOBT was mainly offered by clinicians (48.2%) (Table 1).

**Table 1:** Characteristics of individuals who were offered an iFOBT (n = 920).

Characteristics	Frequency (n)	Percentage (%)	iFOBT testing, n (%)	
			Refused	Accepted
Age (in years)				
<50	115	12.5	218 (28.5)	547 (71.5)
50–74	765	83.2	63 (54.8)	52 (45.2)
≥75	40	4.3	15 (37.5)	25 (62.5)
Gender				
Male	438	47.6	140 (32.0)	298 (68.0)
Female	482	52.4	156 (32.4)	326 (67.6)
Ethnicity				
Malay	727	79.0	241 (33.1)	486 (66.9)
Chinese	113	12.3	40 (35.4)	73 (64.6)
Indian	62	6.7	8 (12.9)	54 (87.1)
Others	18	2.0	7 (38.9)	11 (61.1)
BMI^a^ (in kg/m^2^)				
Underweight (<18.5)	14	1.5	3 (21.4)	11 (78.6)
Normal (18.5–24.9)	407	44.2	137 (33.7)	270 (66.3)
Overweight (25–29.9)	377	41.0	125 (33.2)	252 (66.8)
Obese (≥ 30)	122	13.3	31 (25.4)	91 (74.6)
Education Level				
College/University	41	4.5	21 (28.4)	53 (71.6)
Primary level	284	30.9	11 (26.8)	30 (73.2)
Secondary level	521	56.6	101 (35.6)	183 (64.4)
No formal education	74	8.0	163 (31.3)	358 (68.7)
Occupational status				
Unemployed	551	59.9	160 (29.0)	391 (71.0)
Employed	369	40.1	136 (36.9)	233 (63.1)
Active smokers				
No	720	78.3	212 (29.4)	508 (70.6)
Yes	200	21.7	84 (42.0)	116 (58.0)
Hypertension				
Yes	468	50.9	87 (26.4)	243 (73.6)
No	452	49.1	209 (35.4)	381 (64.6)
Diabetes mellitus				
Yes	349	37.9	74 (21.2)	275 (78.8)
No	571	62.1	222 (38.9)	349 (61.1)
Dyslipidemia				
Yes	233	25.3	48 (21.0)	181 (79.0)
No	687	74.7	248 (35.9)	443 (64.1)
Family history of CRC				
Yes	30	3.3	8 (26.7)	22 (73.3)
No	890	96.7	288 (32.4)	602 (67.6)
Symptomatic				
Yes	38	4.1	6 (15.8)	32 (84.2)
No	882	95.9	290 (32.9)	592 (67.1)
Category of staff offering the test				
Clinician	443	48.2	102 (23.0)	341 (77.0)
Medical assistants	243	26.4	108 (44.4)	135 (55.6)
Nurses	234	25.4	86 (36.8)	148 (63.2)

^a^BMI = Body Mass Index.

Of the patients receiving an offer for the iFOBT, 32.2% (n = 296) refused it. Those without hypertension (adjusted OR: 3.33; 95% CI: 2.44, 4.54) and diabetes mellitus (adjusted OR: 1.99; 95% CI: 1.42, 2.77) were more likely to refuse the test. Furthermore, the absence of CRC-related symptoms was shown to increase their likelihood of refusing the test (adjusted OR: 3.15; 95% CI: 1.26, 7.89). Active smokers were found to have a higher tendency to refuse the iFOBT (adjusted OR: 1.74; 95% CI: 1.22, 2.47). Compared with clinicians, their tendency to refuse the iFOBT was also found to be higher if the offer was made by the medical assistants (adjusted OR: 2.44; 95% CI: 1.71, 3.49) or nurses (adjusted OR: 2.41; 95% CI: 1.65, 3.51) (Table 2).

**Table 2:** Factors associated with the refusal of iFOBT, multiple logistic regression.

	Crude OR^b^	p value	Adjusted OR	p value
	(95% CI)		(95% CI)	
Active smokers				
No	1		1	
Yes	1.74 (1.26, 2.40)	0.001	1.74 (1.22, 2.47)	0.002
Diabetes mellitus				
Yes	1		1	
No	2.36 (1.74, 3.21)	<0.001	1.99 (1.42, 2.77)	<0.001
Hypertension				
Yes	1		1	
No	3.77 (2.80, 5.07)	<0.001	3.33 (2.44, 4.54)	<0.001
Symptomatic				
Yes	1		1	
No	2.61 (1.08, 6.32)	0.03	3.15 (1.26, 7.89)	0.014
Category of staff offering the test				
Physician	1		1	
Medical assistants	2.68 (1.91, 3.74)	<0.001	2.44 (1.71, 3.49)	<0.001
Nurses	1.94 (1.38, 2.75)	<0.001	2.41 (1.65, 3.51)	<0.001

^b^OR = Odds Ratio, significant p value at <0.05;

Crude OR using logistic regression model; Adjusted OR obtained using forward selection method in multiple logistic regression model.

Among the reasons commonly given by those who refused the iFOBT included “feeling not ready for the test” (21.6%), “feeling healthy” (14.9%), “having difficulties with transportation” (7.1%) and “feeling uncomfortable with the test” (3.7%) (Table 3).

**Table 3:** Reasons for refusal of the iFOBT (n = 296).

Reasons	Frequency (n)	Percentage (%)
Feeling not ready for the test	64	21.6
Feeling healthy	44	14.9
Having difficulties with transportation	21	7.1
Having a busy life	10	3.4
Feeling not comfortable with the test	11	3.7
Having the test performed within the last 2 years	6	2.0
No specific reason given	140	47.3

## Discussion

While the CRC screening remains opportunistic in many countries, this study reveals that approximately one-third of the individuals from semi-urban communities have been refusing the iFOBT offered by health care providers. The refusal rate found in this study was higher than that reported in Italy (20%) [27]. It is known that the refusal of CRC screening is strongly associated with the type of screening test selected [28]. Despite being widely perceived as handy and easy to use, the iFOBT was unexpectedly found to be less preferable to colonoscopy in Korea, mainly due to its unhygienic feature [29]. This could partly explain why the uptake of CRC screening is constantly lower than that of breast cancer screening not only in the semi-urban but also in the rural areas [30]. Although patients generally tend to take advices from physicians, it is obvious that the iFOBT screening test is still unacceptable in a considerable proportion of the semi-urban communities [31].

The study finding is in line the Health Belief Model, which links the health behaviours of individuals to their perceived susceptibility, severity, benefits, barriers and cues to action against a particular disease [9,31–33]. The individuals refusing the iFOBT were mainly those who did not have symptoms of CRC (perceived low susceptibility), did not have comorbidities (perceived low severity), were active smokers (perceived low benefits), and had the offers made by nurses and medical assistant recommendations (insufficient cues to action). Through an open-ended question, the common reasons of refusing the iFOBT reported by the included, including ‘feeling not ready’, also point to the perceived low benefits of CRC screening. Therefore, interventions to enhance the iFOBT uptake by modifying their perceptions about CRC screening is warranted.

Although chronic non-communicable diseases, such as diabetes mellitus and hypertension, demonstrated an upward trend in most Asian countries [4], this study shows that the patients who had these two diseases were more likely to accept the offer for the iFOBT. Chronic medical conditions have been associated with a higher risk of cancer [34,35], and it was likely that health care providers had been putting in more efforts to convince the patients with these two diseases to receive the test. It was also likely that the patients with chronic non-communicable diseases were more concerned over their own health conditions.

Even though the early detection of CRC is desirable [36,37], it is noteworthy that the iFOBT had also been occasionally offered to the patients who were outside the recommended age range, as well as to those who presented with presumptive symptoms of CRC. Although such patients should be classified under the “high-risk” category and undergo colonoscopy, it is conceivable that the iFOBT was still offered to them when they expressed their concerns about invasive procedures. As the iFOBT is expected to reduce the cancer burden in the country and the resources in public health institutions are limited, it is important to ensure that the use of the test is well justified. The negative perceptions regarding colonoscopy, particularly in the high-risk groups, also need to be corrected.

Consistent with the previous findings [27,35], the active smokers were also found to be more likely to refuse the iFOBT than non-smokers in this study. In fact, smokers are well known for having a higher risk and mortality rate of CRC [30,38]. This study indicates that the efforts to promote the CRC screening among the smokers might have been insufficient. It also suggests that smokers who have CRC in the semi-urban communities are likely to present late for medical care and thus have a poorer survival in general. Although the refusal to perform the iFOBT could be multifactorial among smokers, including their fear about cancer, it is noted that those who attempted to quit smoking are less likely to refuse the test [39]. Therefore, strategies to upscale the CRC screening in this subgroup is also requited.

Numerous studies found that the incidence of CRC, along with the refusal to perform early screening, is strongly associated with the socioeconomic status of population. Both the incidence and mortality rates also higher in certain ethnic and occupational groups with a lower income and inadequate insurance coverage [40,41]. Meanwhile, the inequality in the participation in cancer screening activities in Spain is linked to the low socioeconomic status of the population [35]. Even though a low socioeconomic status, which is often related to a low education level and limited health literacy, is likely to be one of the major cause of the refusal to perform the CRC screening [22,40,42], the employed group was not found to have differed in their willingness to accept the iFOBT from the unemployed group in this study. Such findings imply that the current model is effective in overcoming the inequality in the CRC screening across populations of different socioeconomic status as in Malaysia.

Efforts to change the negative perceptions about the stool-based screening test, particularly through patient education and strengthening the communication between health care providers and patients, are necessary [43,44]. Nevertheless, this study also shows that the offer for the iFOBT was more likely to be rejected if it was offered by health care providers other than clinicians. Although empowering other health care providers to offer the test to patients could reduce the burden of medical doctors in high-load institutions, the information provided by them to patients could be insufficient, less accurate or less convincing. Thus, going forward, the training to improve the skills required for patient education, should not only focus on medical doctors but also on other health care providers participating in offering the test.

This study has a few limitations. Even though it provides insight into the uptake of the iFOBT in the semi-urban communities, the subsequent actions taken by the respective health institutions following the tests were unclear. Approximately half of the patients also did not specify their reasons of refusing the test, and therefore further investigation with the adoption of qualitative research methods is required.

## Conclusions

This study suggests that the iFOBT was refused by approximately one-third of individuals in semi-urban communities when it was offered to them in public primary care centers. Smoking status and chronic medical conditions, along with the category of health care providers offering the iFOBT, were shown to be associated with their willingness to perform the test. The findings could be used to guide policymakers in developing targeted interventions to promote the early screening and timely treatment of CRC.

## Supporting information

S1 Raw data (XLSX)

S1 File (DOCX)

S2 File (DOCX)
