# Supplementary material for: Rate and associated factors of refusal to perform immunochemical Faecal Occult Blood Test (iFOBT) among semi-urban communities
Source: PLoS One. 2021 Oct 7;16(10):e0258129. doi: 10.1371/journal.pone.0258129 (PMC8496834; doi:10.1371/journal.pone.0258129)
Supplement: S1 File — (DOCX) [file pone.0258129.s002.docx]

| Age: | Occupation : |
| --- | --- |
| Sex: Male Female | Education level: College/University  Ya  Secondary school  Ya  Primary school  Ya  No formal education  Ya |
| Height: Weight: | Smoking status: Yes No  Ya |
| Ethnicity: Malay Chinese India Others | |
| Comorbidities: Diabetes Hypertension Dyslipidemia Others, please state:  Ya | |
| Do you have any sign and symptoms of colorectal cancer? Yes No | |
| Have you been diagnosed with cancer previously : colorectal cancer other types of cancer no | |
| Do you have any positive family history of cancer : colorectal cancer other types of cancer no | |
| Do you agree to perform iFOBT? Yes No | |
| If no, please state why : | |
| iFOBT recommendation performed by: Staff nurse Community nurse  Medical assistant Medical officer  Family Medicine Specialist | |

**COLORECTAL CANCER SCREENING SURVEY FORM**

HEALTH CLINIC :
