# Supplementary material for: Rate and associated factors of refusal to perform immunochemical Faecal Occult Blood Test (iFOBT) among semi-urban communities
Source: PLoS One. 2021 Oct 7;16(10):e0258129. doi: 10.1371/journal.pone.0258129 (PMC8496834; doi:10.1371/journal.pone.0258129)
Supplement: S2 File — (DOCX) [file pone.0258129.s003.docx]

| Umur: | Pekerjaan : |
| --- | --- |
| Jantina : Lelaki Perempuan | Tahap Tertinggi Pendidikan : Kolej/Universiti  Ya  Sekolah Menengah  Ya  Sekolah Rendah  Ya  Tidak Bersekolah  Ya |
| Tinggi : Berat : | Status merokok : Ya Tidak  Ya |
| Bangsa : Melayu Cina India Lain-lain | |
| Sejarah penyakit terdahulu : Diabetes Hipertensi Dislipidemia Lain-lain, nyatakan :  Ya | |
| Adakah klien mengalami tanda dan gejala kanser usus? Ya Tidak | |
| Klien pernah menghidap penyakit kanser sebelum ini : kanser usus kanser lain-lain tidak | |
| Sejarah keluarga klien menghidap penyakit kanser : kanser usus kanser lain-lain tidak | |
| Adakah klien bersetuju untuk menjalani ujian iFOBT ? ya tidak | |
| Jika **tidak**, nyatakan sebab : | |
| Kaunseling iFOBT dilakukan oleh : Jururawat Masyarakat Jururawat Kesihatan  Penolong Pegawai Perubatan Pegawai Perubatan  Pakar Perubatan | |

**BORANG SOAL SELIDIK UJIAN SARINGAN KANSER KOLOREKTAL (USUS)**

KLINIK KESIHATAN :
